# Including Empirical Prior Information in the Reliable Change Index

**DOI:** 10.1177/01466216251358492

**Published:** 2025-07-10

**Authors:** R. Philip Chalmers, Sarah Campbell

**Affiliations:** 1 7991 York University, Toronto, ON, Canada

**Keywords:** reliable change index, IRT, CTT, latent change scores

## Abstract

The reliable change index (RCI; Jacobson & Truax, 1991) is commonly used to assess whether individuals have changed across two measurement occasions, and has seen many augmentations and improvements since its initial conception. In this study, we extend an item response theory version of the RCI presented by Jabrayilov et al. (2016) by including empirical priors in the associated RCI computations whenever group-level differences are quantifiable given post-test response information. Based on a reanalysis and extension of a previous simulation study, we demonstrate that although a small amount of bias is added to the estimates of the latent trait differences when no true change is present, including empirical prior information will generally improve the Type I behavior of the model-based RCI. Consequently, when non-zero changes in the latent trait are present the bias and sampling variability are show to be more favorable than competing estimators, subsequently leading to an increase in power to detect non-zero changes.

## Introduction

Assessing change in psychological research is crucial for evaluating the effectiveness of interventions and understanding individual progress (Maydeu-Olivares, 2021). However, change detection and quantification research often focuses solely on marginal group-level information rather than focusing on individual changes themselves (Blampied, 2022; Wang & Weiss, 2017). In the context of randomized controlled trials (RCTs), for example, the goal of the analysis is generally to obtain estimates of average treatment effects (ATEs) to quantify the causal efficacy of a given intervention program. Although ATEs are useful for detecting and establishing causal relationships, as well for establishing minimally important differences due to causal interventions (Hays & Peipert, 2021), they are less effective at detecting which specific individuals have changed (Hays et al., 2021; Wang & Weiss, 2017). Detecting individual change is further exacerbated in settings where measurement error is non-ignorable, such as in clinical, counseling, medical, personality, and educational measurement settings, where differences are inferred based on less reliable composite scores (Blampied, 2022; Wang & Weiss, 2017).

Detecting whether a given individual has been affected by a causal intervention program is frequently evaluated using person-specific indices, such as the popular reliable change index (RCI; Jacobson & Truax, 1991). The RCI is a measure that explicitly incorporates measurement uncertainty into the inferences on a per-individual basis based on group-level measurement behavior, and has been used to both detect and classify individuals into subsequent categories based on the magnitude of the observed changes. Modifications and extensions of the RCI have frequently appeared in the literature due to the increased demand for better quantification of meaningful changes. For instance, the RCI method has been generalized to include regression to the mean effects (Hageman & Arrindell, 1993; Speer, 1992), which involves pooling measurement errors across occasions when pre-test and post-test data are available, to more sophisticated model-based approaches that do not involve classical test theory (CTT) assumptions (Jabrayilov et al., 2016; Maydeu-Olivares, 2021). When modeling assumptions are reasonably met, model-based approaches are generally more flexible at detecting individual changes than the CTT-RCI formulations in that, for instance, multidimensional constructs can be studied over multiple time points (Wang et al., 2020), and missing data due to dropout or attrition pose less serious issues.

Although focusing on whether, and to what degree, individuals have changed is of primary importance, information regarding whether there are marginal differences across all individuals can still provide useful information in the context of inferring individual differences (Blampied, 2022; Hageman & Arrindell, 1993; Speer, 1992). In the context of item response theory (IRT; Embretson & Reise, 2000) modeling applications for the RCI, however, this topic has yet to be sufficiently explored. Our goal in this article is to therefore investigate and extend the methodology relevant to successful application of the RCI using IRT by including empirical information that utilizes group-level information into the detection and quantification of individual differences. We begin by reviewing the CTT and IRT versions of the RCI, along with their relative strengths and limitations. Following this review a modified version of the IRT-based RCI statistic is presented that includes empirical prior information for Bayesian estimators so that group-level change information can be included directly. A simulation study that replicates and extends the design investigated by Jabrayilov et al. (2016) is then presented to demonstrate the effects and implications of this new approach. The article concludes with a discussion of the strengths and limitations of using empirical priors, and suggests future areas for investigation and improvements.

## Reliable Change Indices Using CTT and IRT

The reliable change index (RCI) presented by Jacobson and Truax (1991), and seen in Equation (1) below, compares two unweighted sum-scores, $X_{\mathrm{post}}$ and $X_{\mathrm{pre}}$ , typically composed from J distinct items on two separate measurement occasions. In clinical contexts, $X_{\mathrm{post}}$ is often taken to be the observed total score for a given individual after therapeutic intervention, where the purpose of the RCI evaluation is to detect whether a given individual has reliably (i.e., “significantly”) changed across the measurement occasions in light of the potential measurement error (Wang et al., 2020). The inclusion of measurement error using CTT reliability information (test-retest reliability, coefficient α for the pre-test or post-test data, etc.) results in the metricless RCI ratio

(1) $${\text{RCI}}_{\text{CTT}}=\frac{X_{\mathrm{post}}-X_{\mathrm{pre}}}{\sqrt{{\mathrm{SE}}^{2}\left(X_{\mathrm{post}}\right)+{\mathrm{SE}}^{2}\left(X_{\mathrm{pre}}\right)}}=\frac{X_{\mathrm{post}}-X_{\mathrm{pre}}}{{\mathrm{SE}}_{d}}$$

where SE represents the standard error of measurement and ${\mathrm{SE}}_{d}$ reflects the standard error of the difference scores (Christensen & Mendoza, 1986; Maydeu-Olivares, 2021). Under the null hypothesis of no change, ${\text{RCI}}_{\text{CTT}}$ is assumed to follow a Gaussian distribution, ${\text{RCI}}_{\text{CTT}}∼N\left(0,1\right)$ , where large-sample confidence intervals are often reported alongside the associated test of significance (Jacobson & Truax, 1991). The associated ${\mathrm{SE}}_{d}$ can be obtained using many approaches, such as via the pooling approach seen in Equation (1), using the SE from only baseline or follow-up test (e.g., internal consistency via coefficient α and ${\mathrm{SE}}_{d}=\sqrt{2\cdot {\mathrm{SE}}^{2}\left(X\right)}$ , which assumes that the SE is constant across testing occasions; Jabrayilov et al., 2016), test-retest reliability estimates (Jacobson & Truax, 1991), factor analytic methods (Claus et al., 2024; Maydeu-Olivares, 2021), among others (Blampied, 2022).

Regardless of the select CTT reliability estimation method, ${\mathrm{SE}}_{d}$ and its sample estimate, ${\hat{\mathrm{SE}}}_{d}$ , are typically taken to be constant across the entire range of the test, which several authors have argued is a principle limitation of the CTT-based RCI (Hays et al., 2021; Stratford et al., 1996). For instance, using simulated response data Jabrayilov et al. (2016) demonstrated that using a constant ${\mathrm{SE}}_{d}$ will often result in measurement uncertainty that is systematically too conservative at the ends of the test’s scoring range, while in the middle of the score distribution ${\mathrm{SE}}_{d}$ will often underestimate the true measurement uncertainty. This leads to conservative detection behavior in the upper and lower ends of the scoring distribution (i.e., true change is detected at a rate lower than the target Type I error rate) and often liberal detection behavior near the center of the score distribution (i.e., ${\text{RCI}}_{\text{CTT}}$ incorrectly suggests that individuals have changed when in fact they have not).

To formally compare the CTT-based RCI to a model-based IRT approach, and to address the expected non-constant behavior of ${\mathrm{SE}}_{d}$ across the range of the test, Jabrayilov et al. (2016) proposed a modification to ${\text{RCI}}_{\text{CTT}}$ that includes estimates of the latent trait $\left(\hat{\theta }\right)$ and their associated large-sample standard errors, $\mathrm{SE}\left(\hat{\theta }\right)$ , of the form

(2) $${\text{RCI}}_{\text{IRT}}=\frac{{\hat{\theta }}_{\mathrm{post}}-{\hat{\theta }}_{\mathrm{pre}}}{\sqrt{{\mathrm{SE}}^{2}\left({\hat{\theta }}_{\mathrm{post}}\right)+{\mathrm{SE}}^{2}\left({\hat{\theta }}_{\mathrm{pre}}\right)}}.$$

In this model-based modification the measurement accuracy of the latent trait is allowed to vary as a function of the test’s measurement capabilities, where it is known that tests have less precision in the ends of the scoring distribution (Lord, 1980). See Finkelman et al. (2010) for an analogous Z -ratio statistic in computerized adaptive testing contexts.

Similar to the selection of which reliability estimator to use for ${\text{RCI}}_{\text{CTT}}$ , one ambiguous area with ${\text{RCI}}_{\text{IRT}}$ involves selecting the estimator for the respective $\hat{\theta }$ and $\mathrm{SE}\left(\hat{\theta }\right)$ terms. Historically common choices for obtaining predictions of θ include the maximum-likelihood criterion (Lord & Novick, 1968), weighted maximum-likelihood estimation (WML; Warm, 1989), expected and maximum a posterior estimates (Bock & Aitkin, 1981), recursive algorithms for obtaining estimates associated with sum-scores (Thissen et al., 1995), among other approaches that focus on varying degrees of inferential and robustness properties (e.g., M-estimators; Wainer & Thissen, 1987). Jabrayilov et al. (2016) utilized the WML^1^ criteria to estimate the associated $\hat{\theta }$ values in Equation (2) due to its unbiasedness when no change is present (Warm, 1989), while the associated $\mathrm{SE}\left(\hat{\theta }\right)$ terms were obtained using the asymptotic behavior of the WML in the form of the Fisher information function, $\mathrm{SE}\left(\theta \right)=\sqrt{I^{-1}\left(\theta \right)}$ . See Hays et al. (2021) and Jabrayilov (2016) for use of the posterior standard deviations when using the expected a posteriori (EAP) estimator.

To compare the efficacy and suitability of ${\text{RCI}}_{\text{CTT}}$ and ${\text{RCI}}_{\text{IRT}}$ , Jabrayilov et al. (2016) evaluated a Monte Carlo simulation experiment that focused on various empirical characteristics. Based on the simulation results, Jabrayilov et al. (2016) concluded that ${\text{RCI}}_{\text{CTT}}$ and the WML version of ${\text{RCI}}_{\text{IRT}}$ demonstrate mixed behavior in terms of Type I error control and power to detect true latent trait changes. In particular, their simulations with five category graded response models (GRMs; Samejima, 1969) revealed that ${\text{RCI}}_{\text{CTT}}$ performed somewhat better for shorter tests (J=5) , however was not universally understood as the best change detection statistic due to its conservative Type I error behavior in the extreme ends of the score distribution and liberal Type I error control near the center of the score distribution. The results of the WML version of the ${\text{RCI}}_{\text{IRT}}$ demonstrated similar power rate difficulties when detecting true latent trait changes, while similar to ${\text{RCI}}_{\text{CTT}}$ had progressively more conservative Type I error control as the θ values became more extreme. Unlike ${\text{RCI}}_{\text{CTT}}$ , however, ${\text{RCI}}_{\text{IRT}}$ did not demonstrate liberal Type I error control behavior across the simulation conditions, and instead tended to approach the nominal α level as the test length increased. This led the authors to conclude that “…IRT is superior to CTT, provided that tests contain, say, at least 20 items” (p. 568). See Keller and Alexandrowicz (2024) for a recent review of similar simulation studies and empirical investigations involving ${\text{RCI}}_{\text{CTT}}$ and ${\text{RCI}}_{\text{IRT}}$ .

## Empirical Prior Information for ${\text{RCI}}_{\text{IRT}}$

By design, the ${\text{RCI}}_{\text{CTT}}$ presented by Jacobson and Truax (1991) focused only on changes between individuals regardless of any group-level changes. However, given how Equation (1) is constructed this position is at odds with how it is applied in practice. That is, for the RCI family, how individual changes are evaluated is directly informed by the group-level response behavior. This is because the statistical information required by the RCI family (e.g., reliability and [pooled] estimates of ${\mathrm{SE}}_{d}$ ) are obtained as a function of the population’s relative change and variability (Wang et al., 2020). Jacobson and Truax (1991) initially took this “individual relative to the group” comparison behavior one step further in their presentation by classifying individuals into (debatably arbitrary) discrete groups as a function of deviations between test score distributions (e.g., relative to the mean and SD of “healthy” and “dysfunctional” groups). While classifications based on a priori score distributions could be seen as contentious, it is evident that knowledge of group-level differences is fundamental in the context of clinical intervention programs (Claus et al., 2024). As we demonstrate in this section, group-level information can—and in many cases, should—be included in the form of Bayesian prior information to provide a useful means to evaluate the degree to which individuals have changed across the measurement occasions.

Bayesian priors have been previously studied in the context of ${\text{RCI}}_{\text{IRT}}$ , first by Jabrayilov (2016) in their unpublished work and later by Hays et al. (2021), where both sets of authors utilized the same informative Gaussian prior distribution (θ∼N(0,1)) when computing both ${\hat{\theta }}_{\mathrm{post}}$ and ${\hat{\theta }}_{\mathrm{pre}}$ in their analyses. However, using the same prior distributions for $\theta_{\mathrm{post}}$ and $\theta_{\mathrm{pre}}$ assumes that individuals have the same population characteristics, where the resulting trait estimates are obtained under the assumption that there is no expected change between measurement occasions. In many applications this assumption is inherently unrealistic as individuals and groups of individuals are frequently expected to change either naturally or via clinical intervention (Claus et al., 2024; McAleavey, 2024). In the case of clinical intervention programs, for example, it is not unreasonable to believe that: $E\left(\theta_{\mathrm{post}}\right)<E\left(\theta_{\mathrm{pre}}\right)$ when the intervention is successful for one or more individuals; the pre-test latent traits have greater variability than the post-test $\left(VAR\left(\theta_{\mathrm{pre}}\right)\geq VAR\left(\theta_{\mathrm{post}}\right)\right)$ due to a wider range of pathology symptoms intensities; individuals simply become less extreme on the latent trait due to natural regression to the mean phenomenon (Hageman & Arrindell, 1993; Speer, 1992); and so on.

In the context of the ${\text{RCI}}_{\text{IRT}}$ , including group-level information appears to have been unexplored despite the potential benefits of including such information (Hageman & Arrindell, 1993; McAleavey, 2024). However, including group-level information that differs from the historically reflexive θ∼N(0,1) prior necessarily changes the concentration of the estimation bias across the possible range of θ . These biases appear in ${\text{RCI}}_{\text{IRT}}$ as the true difference between the pre-test and post-test θ estimates, $\Delta \hat{\theta }={\hat{\theta }}_{\mathrm{post}}-{\hat{\theta }}_{\mathrm{pre}}$ , each of which is shrunk toward the central tendency of the measurement occasion. Ultimately, this results in a bias-variance trade-off based on whether the individual is believed to more closely align with the behavior of the group, or if the individual is evaluated under the assumption that their behavior is independent of the group.

There are three types of bias-variance trade-off situations to consider when applying Bayesian prior distributions in the context of ${\text{RCI}}_{\text{IRT}}$ . The first two situations appear when the prior reflects a sub-optimal match, which occurs when 1) the individual does not change across measurement occasions, but the prior indicates changes given the marginal group behavior, and 2) the individual does change across the measurement occasions, but the prior is organized to reflect no change in the latent trait. Both of these scenarios will result in estimation bias when recovering the true Δθ , which will also negatively impact the overall recovery behavior as quantified by estimators such as the root-mean-square deviation (RMSD). In contrast, when the individual does change across measurement occasions, and so too does the marginal group behavior and resulting empirical prior estimate, then reductions in bias and mean-square error ( MSE ) in $\Delta \hat{\theta }$ should be anticipated, positively impacting the recovery of the true Δθ .

### Estimating and Utilizing Empirical Prior Information

Suppose that a given set of IRT models are fitted to a set of pre-test and post-test response data. The item content and response models themselves do not necessarily need to overlap, as similar information can be extracted via other equating methods (e.g., see Kolen & Brennan, 2004), but for ease of presentation let us assume that the same items appear in both test administrations. To obtain group-level change information the moments, and potentially shape, of the $g\left(\theta_{\mathrm{post}}\right)$ distribution can be estimated relative to moments and shape of the $g\left(\theta_{\mathrm{pre}}\right)$ distribution. This is accomplished by constraining the item parameters to be equal (or even constant) across the respective items over the measurement occasions so that information relevant to $g\left(\theta_{\mathrm{pre}}\right)$ and $g\left(\theta_{\mathrm{post}}\right)$ can be identified and estimated (see Olsbjerg & Christensen, 2015, for details).

Suppose now that a GRM (Samejima, 1969) were fitted to Item A in the pre-test and Item A in the post-test under the constraint that the item parameters are equal, Item B in the pre-test in constrained to be equal to Item B in the post-test, and so on for all relevant item pairings across the testing occasions. This set of constraints reflects the assumption that the test items are invariant across occasions (i.e., do not have differential item or bundle functioning; Chalmers, 2018), but the overt responses differ as a function of the $g\left(\theta_{\mathrm{pre}}\right)$ and $g\left(\theta_{\mathrm{post}}\right)$ . Finally, the metric of the latent traits must be set, which for the pre-test is commonly fixed to a 0-1 scaling when the maximum entropy Gaussian distribution is adopted $\left(\theta_{\mathrm{pre}}∼N\left(0,1\right)\right)$ while for the post-test the moments in a Gaussian distribution can vary to account for the potential mean and variance shifts (e.g., $\theta_{\mathrm{post}}∼N\left(\mu_{\mathrm{post}},\sigma_{\mathrm{post}}^{2}\right)$ ; Olsbjerg & Christensen, 2015). If Gaussian distributions for θ are less plausible then the shape of the pre-test or post-test g(θ) distribution may be modeled using other means (e.g., via empirical histograms; Woods, 2007), though for our presentation we assume Gaussians are sufficient.

Including the shape and moment information in $g\left(\theta_{\mathrm{post}}\right)$ (and potentially in $g\left(\theta_{\mathrm{prior}}\right)$ ) ultimately results in adding empirical prior information for the $\hat{\theta }$ predictions, systematically shrinking the predictions to the group’s expected behavior, which in RCI applications can be understood in a positive light (Speer, 1992). Specifically, if the group is generally changing then it is more likely that any given individual will have changed as well, provided that this is supported by their overt response pattern. With this potential augmentation in mind, Equation (2) may be reorganized such that components associated with ${\hat{\theta }}_{\mathrm{pre}}$ are estimated using Bayesian methods that incorporate $g\left(\theta_{\mathrm{pre}}\right)$ (e.g., EAP and MAP) given the calibrated pre-test IRT model, which in the following application we set to $\theta_{\mathrm{pre}}∼N\left(0,1\right)$ . The components that pertain to ${\hat{\theta }}_{\mathrm{post}}$ , on the other hand, can obtained using an IRT model with the same item parameters as the pre-test but with an updated $g\left(\theta_{\mathrm{post}}\right)$ such as $\theta_{\mathrm{post}}∼N\left(\mu_{\mathrm{post}},\sigma_{\mathrm{post}}^{2}\right)$ , or in practice $\theta_{\mathrm{post}}∼N\left({\hat{\mu }}_{\mathrm{post}},{\hat{\sigma }}_{\mathrm{post}}^{2}\right)$ . See Appendix A for an example of how this type of analysis can be evaluated using code from the mirt package (Chalmers, 2012).

## Simulation Study

Including group-level effects using Bayesian estimators is only useful in RCI applications insofar as the associated group-level shrinkage does not invoke substantial bias in the subsequent inferences, and in principle should not result in sub-optimal false positive detection behavior for ${\text{RCI}}_{\text{IRT}}$ . As such, simulation experiments should be explored to evaluate whether the inclusion of empirical prior information negatively affects the Type I error behavior, power rates, and associated parameter inferences quantifying the individual change estimates, $\Delta \theta =\theta_{\mathrm{post}}-\theta_{\mathrm{pre}}$ .

Rather than designing a new set of Monte Carlo simulation conditions we instead chose to replicate and extend the GRM simulation found in Jabrayilov et al. (2016). The reason for adopting this simulation design was to reevaluate whether the authors’ original recommendation to use ${\text{RCI}}_{\text{IRT}}$ only with 20 or more items would remain when using the EAP estimator with group informed empirical priors, and to evaluate whether ${\text{RCI}}_{\text{CTT}}$ would remain a preferred method in tests containing only five items. The complete set of fully crossed conditions found in Jabrayilov et al. (2016) pertaining to three test lengths (J=5,10,20) , four magnitudes of Δθ (Δθ=[0,−1/2,−1,−1.5]) , range of conditional θ values ( −2.5 to 3.5 in steps of .05, each evaluated over R=100 replications, within which N=5000 response vectors were sampled and evaluated), and two correctly specified item generation sets (homogeneous vs. heterogeneous) were investigated.

The homogeneous item sets for the GRM were randomly sampled within each replicate, where the discrimination parameters were sampled from a uniform distribution with the range [1.5,2.5] while the four difficulty parameters were sampled from $\left[-1,-0.5,0.5,1\right]+\bar{b}$ , where $\bar{b}$ was sampled from the uniform distribution with [0,1.25]. For the heterogeneous item sets the discrimination parameters were sampled from a uniform distribution with the range [1,2.5] while the four difficulty parameters were sampled from $\left[-0.5,-0.2,0.2,0.5\right]+\bar{b}$ , where $\bar{b}$ was sampled from the uniform distribution with [−1.5,2.5] . The homogeneous item sets represents empirical scenerios where symptom traits are within a more narrow θ range, while the heterogenous item sets are reflect applicationsthat have a wider θ range (see Jabrayilov et al., 2016, for further explanation). Population coefficient α (and their resulting ${\mathrm{SE}}_{d}$ ) values were estimated from these item parameter sets after generating response data from a “clinical” population where θ∼N(1/2,1) (see Jabrayilov et al., 2016), resulting in the estimates [.829,.906,.951] for the homogeneous item parameter sets and [.676,.809,.895] for the heterogeneous item parameter sets across the three respective test lengths.

Of note, the “clinical population” design choice used by Jabrayilov et al. (2016) makes the comparison of the CTT and IRT approaches more difficult in its current form as the resulting reliability and precision information are on different θ scales. In the current form, ${\text{RCI}}_{\text{CTT}}$ obtains the requisite ${\mathrm{SE}}_{d}$ information from item parameters that have been calibrated on a clinical population with θ∼N(1/2,1) , while the IRT model assumes that the item parameters have been calibrated on a clinical population with θ∼N(0,1) . Had the IRT model been calibrated on the hypothetical θ∼N(1/2,1) population the resulting GRM difficulty terms would have necessarily been shifted to lower values when the canonical 0-1 scaling for θ is retained. Placing the IRT and CTT models on the same metric could be amended by either a) retaining the canonical θ∼N(0,1) assumption in the IRT model but shifting the difficulty parameters through item parameter linking (Kolen & Brennan, 2004)^2^, or b) for the ${\text{RCI}}_{\text{CTT}}$ approach simply assume the clinical population is centered at the mean of θ=0 as the θ scale is generally arbitrary. In either case, in future studies we recommend that comparisons involving CTT and IRT methods be placed on the same θ scale, though in the spirit of replicability we have remained consistent with the original simulation structure as our focus primarily pertains to comparing suitably scaled IRT methods rather than the ${\text{RCI}}_{\text{CTT}}$ with a different scale.

For the purpose of this investigation, Jabrayilov et al.’s (2016) simulation design was extended by including EAP estimators with varying group-level informed prior distribution concentrations. Specifically, an informative prior reflecting no group-level changes was included, as well as approximate small, medium, and large group-level changes over measurement occasions, expressed in terms of Cohen’s (1988) standardized d metric^3^. For all EAP conditions we assumed that $\theta_{\mathrm{pre}}∼N\left(0,1\right)$ , which is often the canonical assumption for IRT models that require 0-1 scaling (Embretson & Reise, 2000), while for $\theta_{\mathrm{post}}$ we assumed gradually increasing group-level differences in the form of $\mu_{\mathrm{post}}$ between the pre-test and post-test traits; namely, N(0,1) , N(−0.2,1) , N(−0.5,1) , N(−0.8,1) , the first of which matches the informative “no expected change” prior found in Jabrayilov (2016) and Hays et al. (2021). In practice, such latent variable effects would need to be estimated from suitable empirical data, and in some cases alternative latent trait distribution shapes may be required whenever Gaussian distributions are deemed unsuitable. Note that the negative $\mu_{\mathrm{post}}$ values are intended to reflect a positive change for the group as the RCI methods have primarily been applied to psychological instruments measuring some degree of psychopathy, where lower trait estimates post-intervention reflect lower severity on said trait.

The simulation experiment was evaluated using open-sourced software in R (R Core Team, 2024) with the SimDesign package (Chalmers & Adkins, 2020) to control the code-base and execution and the mirt package (Chalmers, 2012) for all IRT modeling and prediction components. For the complete simulation code and results see the files stored in the following OSF link: https://osf.io/jnv64/?view_only=5ff138d707734c6989ac0605b4a0d74b

### Marginal Parameter Recovery Results

To highlight the benefits and consequences of including empirical priors with competing highest probability density regions, Table 1 presents the WML and EAP recovery of the true Δθ effects in terms of mean absolute deviations, $E\left(\Delta \theta -\Delta \hat{\theta }\right)$ , averaged over the entire θ range and item parameter sets. In general, unbiased estimates of the Δθ were obtained for the WML and ${\text{EAP}}_{N\left(0,1\right)}$ estimators when there was no change present, which is expected given the nature of the estimators and prior belief specifications (see Warm, 1989). Within the same Δθ=0 conditions, the EAP estimators with non-zero d effects demonstrated small degrees of bias when quantifying the difference, which is also expected given that the prior density is concentrated around $\mu_{\mathrm{post}}$ rather than 0. Note that the strength of this bias decreased as the test length increased, and in theory will decrease to 0 as the test length increases indefinitely.

**Table 1.** Marginal bias estimates for the $\Delta \theta -\Delta \hat{\theta }$ accuracy, presented with respect to the test length and true Δθ conditions.

Conditions		Estimators				
Δθ	J	WML	${\text{EAP}}_{N\left(0,1\right)}$	${\text{EAP}}_{N\left(-0.2,1\right)}$	${\text{EAP}}_{N\left(-0.5,1\right)}$	${\text{EAP}}_{N\left(-0.8,1\right)}$
−1.5	5	0.42	0.65	0.59	0.50	0.40
	10	0.29	0.49	0.45	0.39	0.32
	20	0.19	0.35	0.33	0.28	0.24
−1	5	0.26	0.42	0.36	0.27	0.18
	10	0.16	0.30	0.27	0.21	0.15
	20	0.09	0.21	0.19	0.15	0.11
−0.5	5	0.12	0.21	0.15	0.07	−0.02
	10	0.07	0.15	0.11	0.06	0.00
	20	0.04	0.10	0.08	0.04	0.01
0	5	0.00	0.00	−0.05	−0.14	−0.22
	10	0.00	0.00	−0.03	−0.08	−0.14
	20	0.00	0.00	−0.02	−0.05	−0.08

The Δθ<0 conditions, on the other hand, demonstrated more interesting marginal bias behavior in that the strength of the Δθ effect was more strongly influenced by the informative prior distributions. Specifically, the closer the Δθ effect was to the $\mu_{\mathrm{post}}$ parameter the lower the bias, where a best-case scenario occurred when ${\text{EAP}}_{N\left(-0.5,1\right)}$ was used with Δθ=−0.5 as the estimator’s highest prior density for the post-test was located exactly at the true change magnitude. The net effect of including alternative prior weights in this case is evident: in terms of bias, the ${\text{EAP}}_{N\left(0,1\right)}$ and ${\text{EAP}}_{N\left(-0.2,1\right)}$ priors generally appear worse than the WML whenever Δθ≠0 , however when stronger $\mu_{\mathrm{post}}$ values are included the bias tends to becomes lower than the bias in the WML estimator. As such, when true changes are present the WML may not be the most optimal estimator in terms of bias when informative empirical prior distribution information is available.

Similar results were discovered when investigating the RMSD behavior of Δθ across estimators. As seen in Table 2, the inclusion of an informative prior has the universal effect of reducing the magnitude of RMSD, reflecting greater recovery precision even in the presence of the bias witnessed in Table 1. While increasing the test length consistently reduced the RMSD for all estimators, the effect was more prominent when the studied Δθ were closer to the $\mu_{\mathrm{post}}$ location. Decreasing the value of $\mu_{\mathrm{post}}$ also tended to reduce the RMSD whenever the change was anything other than 0; in the Δθ=0 condition more extreme $\mu_{\mathrm{post}}$ values resulted in gradually worse RMSD behavior, though the marginal recovery remained more optimal than WML in each condition studied due to the smaller MSE . In general, the EAP estimates had consistently lower RMSD values than WML whenever $\mu_{\mathrm{post}}$ was less than 0, again highlighting the potential parameter recovery benefits when including empirical prior information.

**Table 2.** Marginal RMSD estimates presented with respect to the test length and Δθ conditions. Lowest RMSD values per condition are presented in bold font.

Conditions		Estimators				
Δθ	J	WML	${\text{EAP}}_{N\left(0,1\right)}$	${\text{EAP}}_{N\left(-0.2,1\right)}$	${\text{EAP}}_{N\left(-0.5,1\right)}$	${\text{EAP}}_{N\left(-0.8,1\right)}$
−1.5	5	0.94	0.88	0.84	0.78	**0.73**
	10	0.77	0.71	0.69	0.65	**0.62**
	20	0.61	0.56	0.54	0.52	**0.50**
−1	5	0.80	0.71	0.67	0.63	**0.60**
	10	0.65	0.58	0.56	0.54	**0.52**
	20	0.52	0.46	0.45	0.43	**0.42**
−0.5	5	0.71	0.57	0.55	0.53	**0.53**
	10	0.58	0.49	0.47	0.46	**0.46**
	20	0.46	0.39	0.39	0.38	**0.38**
0	5	0.67	**0.50**	0.51	0.53	0.57
	10	0.56	**0.44**	0.45	0.46	0.47
	20	0.45	**0.37**	0.37	0.38	0.38

### Type I Error Results

This section focuses on the rejection behavior in the Δθ=0 conditions to investigate how frequently the null hypothesis of no change is empirically rejected at the level α=.10 . Jabrayilov et al. (2016) and Maydeu-Olivares (2021) demonstrated that the Type I error behavior is generally sub-optimal in regions where the test’s information function is low, where, for instance, detection often becomes more conservative as |θ| tends to the extremes. Jabrayilov et al. (2016) also observed that the CTT version of RCI tended to have more liberal Type I error behavior in the middle of the θ range for their homogeneous item generation conditions regardless of the test length studied, while the WML estimator tended to have nominal to conservative Type I error behavior across all conditions studied.

**Figure 1.**
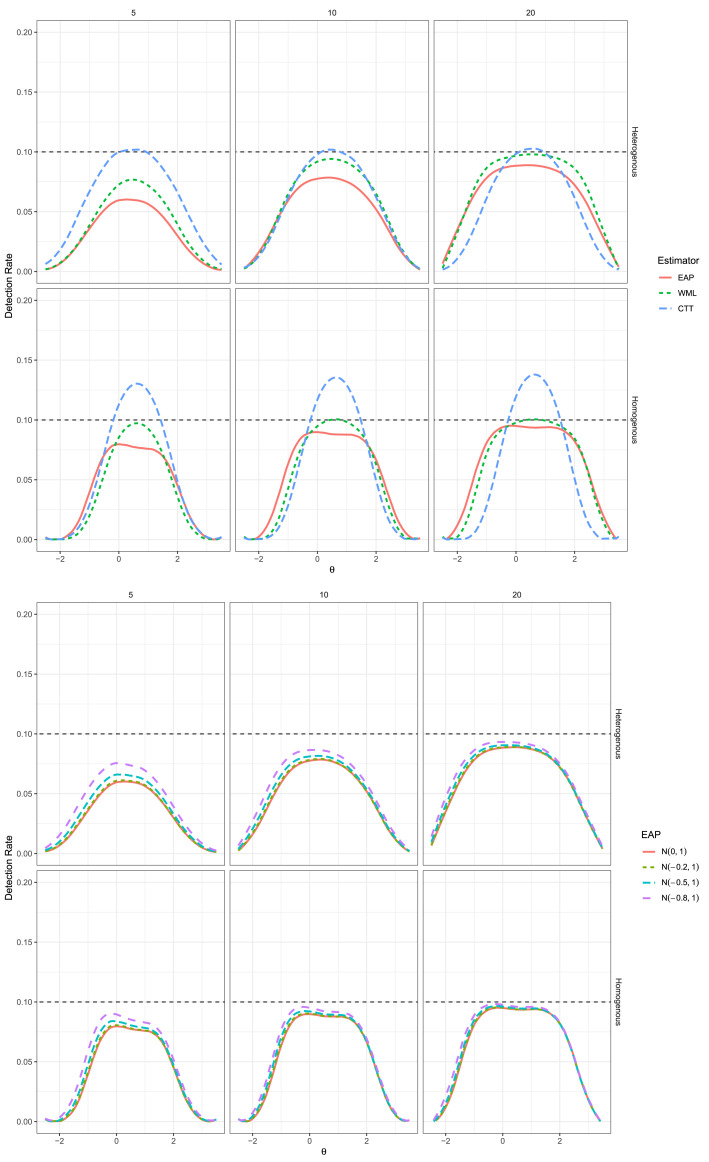
Type I Error Rates for CTT and IRT (EAP and WML) Versions of RCI in the Top Plot, With Bottom Plot Indicating the EAP Estimator With Different Priors for $\theta_{\mathrm{post}}$ . Solid Red Line Corresponds to EAP With N(0,1) Prior in all Images, while the Dashed Black Line Indicates the Nominal α=.10 Rate. Rows Correspond to the Item Generation Design (Heterogeneous vs. Homogeneous) while Columns Indicate the Test Length.

Our replication and extension of these Type I error findings can be seen in the top of Figure 1, which agrees closely with Figure 2 in Jabrayilov et al. (2016, p. 567).^4^ In particular, the WML estimator indeed demonstrated more optimal Type I error control than ${\text{EAP}}_{N\left(0,1\right)}$ in the heterogeneous item set condition, as well as in the middle of the homogeneous item set conditions, while both latent trait estimators demonstrated progressively more optimal rejection behavior as the test length increased. As well, ${\text{RCI}}_{\text{CTT}}$ demonstrated reasonable Type I error rate control in the heterogenous item set conditions, being either at or slightly below the nominal α , while in the homogenous item set conditions demonstrated liberal Type I error control that peaks at the location θ=1/2 N(1/2,1) .

**Figure 2.**
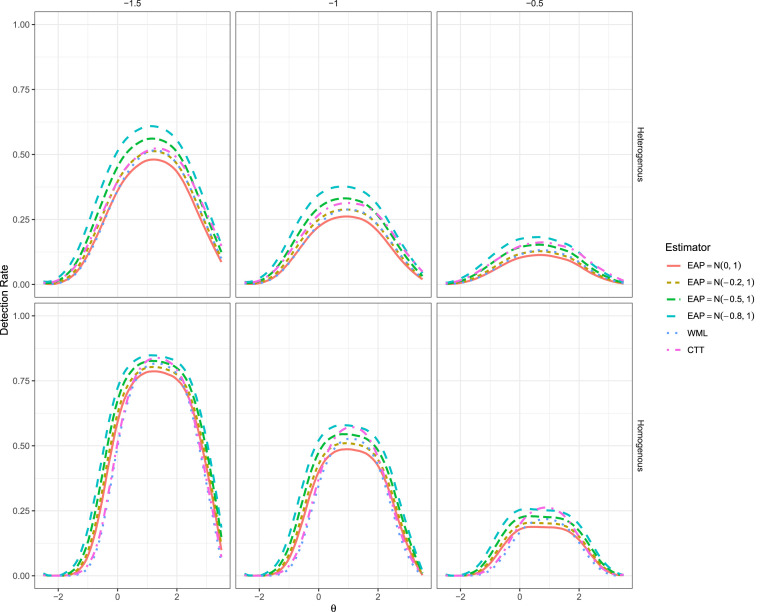
Power Rates for Five Item Test With $\Delta \theta =\theta_{\mathrm{post}}-\theta_{\mathrm{pre}}$ Changes Organized by Column and Item Format (Heterogeneous vs. Homogeneous) by Row.

To add to these results, the bottom set of plots in Figure 1 demonstrate the effect of varying $\mu_{\mathrm{post}}$ locations for the EAP estimator relative to the often reflexive—yet nevertheless informative— $\theta_{\mathrm{post}}∼N\left(0,1\right)$ prior distribution. What is apparent from these plots is that the Type I error rates remained slightly conservative in the full θ range regardless of the $\mu_{\mathrm{post}}$ value, however the rates appeared to be slightly more favorable in that as the magnitude of $\mu_{\mathrm{post}}$ increased the detection behavior became marginally closer to the nominal level across the full range of θ . Most importantly in this context is that even under different Gaussian prior concentrations the EAP estimator’s Type I error behavior remained conservative over the full range of θ , and therefore did not have detrimental effects on the Type I error behavior of the EAP estimator overall.

### Power Rate Results

**Figure 3.**
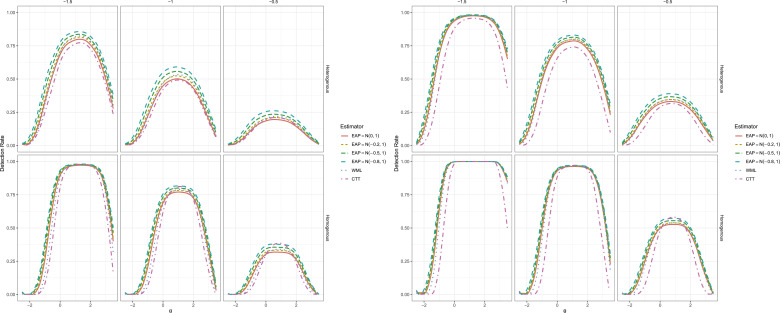
Power Rates for Ten Item (Left Set) and Twenty Item (Right Set) Tests.

While the Type I error behavior only marginally improved after modifying the prior distribution concentrations, the effect of including the group-level empirical prior information had a much larger impact when in fact there were non-zero changes present; see Figures 2 and 3. Beginning with the 5-item test, even with the slightly more conservative detection behavior the EAP estimators with $\mu_{\mathrm{post}}<0$ frequently demonstrated better power to detect non-zero Δθ changes across the studied θ range, where larger Δθ effects were detected with higher power across the full range of θ . Notably, the power of ${\text{EAP}}_{N\left(-0.8,1\right)}$ was in some cases more than 10% higher than both the CTT and WML estimators despite its slightly more conservative detection behavior, though the differences became less pronounced as θ tended to the extremes.

In the homogeneous item set conditions ${\text{RCI}}_{\text{CTT}}$ appeared to demonstrate higher power than the empirical prior versions of EAP in a narrow θ range around θ=1/25 (i.e., the mean of the clinical population), and therefore could be misinterpreted as the most powerful estimator within this range. However, the power behavior observed was largely a function of the inflated Type I error behavior around the location θ=1/2 , and therefore is not an accurate reflection of the ${\text{RCI}}_{\text{CTT}}$ ’s practical utility unless a more strict α criterion were used in this θ range (see Jabrayilov et al., 2016, for further discussion).

Finally, as was observed in Jabrayilov et al. (2016) and replicated in this simulation, all IRT estimators tended to become more powerful as the test length increased (see Figure 3), generally outperforming the CTT version of RCI by increasingly wide margins—excluding, of course, the θ range in the homogeneous item set conditions where ${\text{RCI}}_{\text{CTT}}$ was inflated due to sub-optimal Type I error control. Moreover, higher values of $\mu_{\mathrm{post}}$ in the EAP estimator resulted in progressively higher power across the full range of θ due to the regression to the group mean effects. The WML estimator tended to lie somewhere in the middle of the non-zero $\mu_{\mathrm{post}}$ EAP estimators and the CTT results, and often demonstrated detection behavior similar to that of ${\text{EAP}}_{N\left(-0.2,1\right)}$ .

## Discussion

This article proposed a simple yet potentially beneficial augmentation to the ${\text{RCI}}_{\text{IRT}}$ involving a set of Bayesian estimators previously thought to be sub-optimal. In our extension of the simulation presented Jabrayilov et al. (2016), we demonstrated that regardless of the test lengths studied the EAP estimator can provide competitive bias and RMSD behavior under different Gaussian density configurations, particularly in circumstances where group-level latent change effects are present. In conditions where there was no change in the latent traits across measurement occasions small degrees of bias in the change score estimates were observed whenever the group-level effects were included in the prior, however this bias did not result in sub-optimal null hypothesis rejection behavior according to the empirical Type I error results. Power to detect non-zero changes, on the other hand, was notably higher than previously investigated estimators when including group-level change information in the EAP priors, and the RMSD recovery behavior of the true latent trait changes was universally lower for the EAP estimators compared to the WML estimator.

Based on our observations, we recommend using EAP-based estimators whenever there are meaningful group-level differences across the measurement occasions, whether natural (e.g., regression to the mean; Speer, 1992) or due to clinical interventions, as borrowing strength from marginal group-level information naturally leads to improved inferences regarding individual change behavior. As we demonstrated, including additional empirical information, regardless of the number of test items, will not only improve the precision of the difference between individuals on separate measurement occasions but will ultimately help detect and quantify the degree to which an individual has changed in light of the model-implied measurement uncertainty. In circumstances where there are no group-level effects present then using the non-informative WML approach remains a useful strategy to evaluate the $\theta_{\mathrm{post}}-\theta_{\mathrm{pre}}$ differences, at least until a suitable set of post-test response information becomes available.

With respect to practical application of ${\text{RCI}}_{\text{IRT}}$ , as well as the augmented EAP extension presented herein, the statistics unfortunately require dedicated computer programs to estimate the trait values and their associated measurement errors by supplying an individual’s response vector pattern in its entirety. In contrast, ${\text{RCI}}_{\text{CTT}}$ is notably easier to utilize in applied settings as only sum-score differences and a single ${\mathrm{SE}}_{d}$ are required. This ease of computation is attractive for clinicians as evaluating Equation (1) by hand or mental calculation is trivial. While interactive GUI software do exist for ${\text{RCI}}_{\text{IRT}}$ scoring, such as within the mirt package’s RCI() function, manually supplying complete response vectors may be seen are too onerous when longer tests are used.

That said, it remains possible to use ${\text{RCI}}_{\text{IRT }}$ with EAP-based estimators in the same convenient manner as the ${\text{RCI}}_{\text{CTT}}$ whenever sum-scores are preferred. This can be accomplished by adopting the recursive EAP-based θ estimates associated with the test’s sum-scores using the algorithm described in Thissen et al. (1995). While slightly less optimal than using the complete response vector, the $\hat{\theta }$ and posterior SE estimates associated with the EAP for sum-scores method can naturally be substituted into Equation (2) without any loss of generality, thereby addressing the sub-optimal use of a constant ${\mathrm{SE}}_{d}$ in RCI for all test score differences (Jabrayilov et al., 2016) while simultaneously allowing for easier hand or mental calculation by the clinician. Most notably for this presentation, the augmented ${\text{RCI}}_{\text{IRT }}$ herein applies equally well in this sum-score context, where θ estimates with and without the group-level information can be incorporated into suitable “look-up” tables for convenience. Though not presented in the simulation above, the EAP for sum-scores estimator was highly similar to the EAP estimator’s results, and therefore can provide a practical EAP-based alternative whenever supplying sum-score information is more convenient than supplying response vectors in their entirety.

### Limitations and Future Directions

Although there are benefits to including group-level information in the RCI statistics, there are a number of requirements for this approach to be justifiable in practice. Specifically, to apply the augmented version of ${\text{RCI}}_{\text{IRT }}$ both the pre-test and post-test response data must be jointly available so that the item parameters and group-level change information can be obtained^5^. When only pre-test response data are available the IRT model parameters may be calibrated as usual, however any group-level change information will be unavailable until after suitable post-test response data becomes available. In this situation, WML estimates may be attractive to adopt given its use of the uninformative prior distribution, at least temporarily, though after a sufficient amount of post-test response information becomes available opportunities for empirical prior-based improvements may be made.

Regarding the use of priors that are more information that the non-informative Jeffrey’s prior used in the WML estimation, there are some noteworthy risks that should also be considered. For example, if an individual’s trait were to deteriorate post-intervention $\left(\theta_{\mathrm{post}}>\theta_{\mathrm{pre}}\right)$ while the group tended to improve on average (e.g., $E\left(\theta_{\mathrm{post}}\right)<E\left(\theta_{\mathrm{pre}}\right)$ ) the individual’s atypical change will be regress toward the group’s central tendencies, potentially diminishing or—in extreme cases—masking the atypical change; this issue remains true in augmented versions of ${\text{RCI}}_{\text{CTT }}$ as well (Speer, 1992). To help circumvent this issue, it may therefore be advantageous to compute uninformative Δθ estimates alongside the augmented EAP estimates for individuals who appear to behave differently than the group, or who are more likely to have been unaffected by the clinical intervention. Relatedly, if common causes for the atypical recovery behavior can be discerned and subsequently modeled, such as via regression, mixture, or multi-group estimation methods, then such predictive information should be included in place of the single empirical prior distribution utilized in this presentation (Wang et al., 2023).

Finally, our extended simulation study omits many empirical and model-based properties that should be investigated in future applications. In particular, the information presented herein, as well as in Jabrayilov et al. (2016), are based on fixed and known population-level characteristics using the (correctly specified) graded response model and Gaussian distributed θ terms. IRT modeling, however, is generally considered a large-sample method, and therefore may be less useful if the sample sizes available for parameter estimation are insufficient, and can provide sub-optimal or flawed inferences if the models are unreasonably misspecified. Future work should investigate when the ${\text{RCI}}_{\text{IRT}}$ approach becomes practically consistent in finite sample size conditions, and with alternative item response functions and θ distributions, so that calibration recommendations in the RCI context can be made. This will be particularly important whenever ${\text{RCI}}_{\text{IRT}}$ is applied in more complex applications, such as those involving multiple time points or multidimensional models (Wang & Weiss, 2017)*,* when the shape of the g(θ) distributions significantly deviates from the maximum entropy Gaussian distribution (Woods, 2006, 2007), as well as when applying minimum (clinically) important difference criteria (Jayadevappa et al., 2017; Terwee et al., 2021) with or without equivalence testing criteria (Wellek, 2010).

## Supplemental Material

Supplemental Material - Including Empirical Prior Information in the Reliable Change Index Supplemental Material for Including Empirical Prior Information in the Reliable Change Index by R. Philip Chalmers, and Sarah Campbell in Applied Psychological Measurement

## Notes

1. The WML criteria is equivalent to Bayesian maximum a posteriori estimate using the uninformative, transformation invariant Jeffreys prior.

2. For EAP/MAP estimators, an equivalent would be to set the prior to θ∼N(1/2,1) and retaining the original item generating sets in order to account for the θ location shift. For ML and WML estimators, however, only parameter linking would be appropriate.

3. The d group-level effect size is obtained via $d=\frac{E\left(\theta_{\mathrm{post}}\right)-E\left(\theta_{\mathrm{pre}}\right)}{\sigma_{\theta }}$ , where $\sigma_{\theta }$ is the pooled standard deviation; however, in this study all variances were fixed to 1 and therefore can be understood more simply as $d=E\left(\theta_{\mathrm{post}}\right)-E\left(\theta_{\mathrm{pre}}\right)$ .

4. Similar to Jabrayilov et al. (2016) all figures in this article are presented using locally estimated scatterplot smoothing (LOESS) lines for less noisy presentation of the detection behavior across the select θ range.

5. See Hageman and Arrindell (1993) for an analogous limitation in the context of ${\text{RCI}}_{\text{CTT}}$ , as well as applications of ${\text{RCI}}_{\text{CTT}}$ that pool the associated ${\mathrm{SE}}_{d}$ estimate.
