# Supplementary material for: Including Empirical Prior Information in the Reliable Change Index
Source: Appl Psychol Meas. 2025 Jul 10;49(8):460–76. doi: 10.1177/01466216251358492 (PMC12245826; doi:10.1177/01466216251358492)
Supplement: Supplemental Material - Including Empirical Prior Information in the Reliable Change Index [file sj-pdf-1-apm-10.1177_01466216251358492.pdf]

Appendix

Example Code for RCI_{IRT} Using the `mirt` Package

This appendix demonstrates how the μ_{post} and σ^2_{post} terms can be estimated from a set of suitable data. For practical purposes we provide a set of simulated data given the $\mu_{post} = -0.5$, $J = 20$, heterogeneous item set simulation conditions described in the text with $N = 2000$ response vectors for the pre-test and post-test. All individuals were organized to have a change of $\Delta\theta = -0.5$ across the pretest and posttest administrations, though only the first three response patterns are analyzed and presented below.

```
library(mirt)
set.seed(42)

# generate item parameters for GRM
test_length <- 20
as <- runif(test_length, 1, 2.5)
bbar <- runif(test_length, -1.5, 2.5)
bs <- cbind(bbar - 0.5, bbar -0.2, bbar + 0.2, bbar + 0.5)

# convert to slope-intercept form for mirt
pars <- t(sapply(1L:test_length, \(i)
          traditional2mirt(c(a1=as[i], b=bs[i,]),
                             cls='graded', ncat=5)))

# response data
Theta_pre <- rnorm(2000)
Theta_post <- Theta_pre - 0.5
predat <- simdata(a=pars[,1], d=pars[,-1], itemtype='graded',
                  Theta=Theta_pre)
postdat <- simdata(a=pars[,1], d=pars[,-1], itemtype='graded',
                   Theta=Theta_post)
```

After having access to these two sets of sample data there are now multiple approaches to obtaining estimates of μ_{post} and σ^2_{post}, such as combining the data and estimating a correlated two-dimensional IRT model with cross-factor constraints for each respective item (Keller & Alexandrowicz, 2024), or by simply estimating the μ_{post} and σ^2_{post} assuming that the item parameters from the pretest are provisionally fixed and known. We demonstrate the latter

approach below using a two-step marginal maximum-likelihood (MML) estimation approach.

```
mod_pre <- mirt(predat, itemtype = 'graded')
sv <- mod2values(mod_pre)
sv$est <- FALSE                              # fix all estimates
sv$est[sv$name == 'MEAN_1'] <- TRUE          # estimate mean
sv$est[sv$name == 'COV_11'] <- TRUE          # estimate variance

mod_post <- mirt(postdat, itemtype='graded', pars=sv, verbose=FALSE)
coef(mod_post, simplify=TRUE)[c("means", "cov")]

## $means
##         F1
## -0.4922715
##
## $cov
##           F1
## F1 0.9863365
```

In the above R code a set of GRMs are fitted to each respective item using the MML criteria given the first time point dataset (`predat`), after which an object containing the estimated parameters and their properties are constructed (the object `sv`). This object is then repurposed to force the item parameter estimates to be fixed at the MML estimates, and the latent mean and variance of the assumed Gaussian distribution for θ is are set as estimable parameters. This model structure is that used on the `postdat` to obtain $\hat{\mu}_{post}$ and $\hat{\sigma}^2_{post}$ and subsequently printed.

Given these model specifications the function `RCI()` in the `mirt` package (Chalmers, 2012) can be used to evaluate all RCI computations, including RCI_{CTT}. The output below presents two versions of RCI-IRT with the EAP estimator, the first of which assumes that the θ_{pre} and θ_{post} follow a $N(0,1)$ structure, while the second analysis assumes $\theta_{pre} \sim N(0,1)$ and the empirical prior estimate $\theta_{post} \sim N(\hat{\mu}_{post}, \hat{\sigma}^2_{post})$. In the output, `pre.score` and `post.score` reflect the respective θ_{pre} and θ_{post} estimates, whether these estimates successfully `converged` or not if the estimator required iterative numerical optimization methods (not relevant for EAP estimates), the estimated `diff`-erence $\hat{\theta}_{post} - \hat{\theta}_{pre}$, and finally the inferential information pertaining to the RCI-IRT method in the form of the standard error, large-sample z statistic and

resulting p-value under the null of no latent trait change, respectively.

```
# EAP with theta ~ N(0,1) for pre and post data
RCI(mod_pre, predat=predat[1:3,], postdat=postdat[1:3,])

##   pre.score post.score converged   diff    SE      z     p
## 1    -0.068     -0.300      TRUE -0.232 0.401 -0.578 0.563
## 2    -1.473     -1.622      TRUE -0.149 0.514 -0.290 0.772
## 3    -0.118     -1.026      TRUE -0.908 0.433 -2.098 0.036

# EAP with theta_pre ~ N(0, 1) and theta_post ~ N(mu_hat, sigma2_hat)
RCI(mod_pre, predat=predat[1:3,], postdat=postdat[1:3,],
    mod_post=mod_post)

##   pre.score post.score converged   diff    SE      z     p
## 1    -0.068     -0.339      TRUE -0.271 0.402 -0.675   0.5
## 2    -1.473     -1.689      TRUE -0.216 0.519 -0.416 0.677
## 3    -0.118     -1.079      TRUE -0.961 0.435 -2.209 0.027
```

Finally, and for posterity, the above IRT-based output can be compared to RCI-CTT if suitable estimates of the marginal reliability are available. Supposed that `predat` represented a sufficient sample to obtain a reliability estimates for the respective composite scores, in which case the following code could be used to obtain the associated $\widehat{SE}_d$ estimates using the coefficient α estimate. The results of the following RCI-CTT output are similar to the above RCI-IRT, however note that `pre.score`, `post.score`, and `diff` now represent the sum-scores and their respective difference for each individual, and that `SE` is now a constant. As well, note that the RCI-IRT methods generally result in higher z and lower p-values than the RCI-CTT approach, generally reflecting the higher power to detect latent trait differences (unless RCI-CTT were to display liberal Type I error control behavior, in which case the converse could artificially be true).

```
# descriptive and inferential item statistics for predat
(teststats <- itemstats(predat)$overall)

##     N mean_total.score sd_total.score ave.r  sd.r alpha SEM.alpha
##  2000           27.427         14.415 0.275 0.092 0.881     4.968
```

```r
# RCI-CTT. By default assumes that SEM.pre == SEM.post
RCI(predat = predat[1:3,], postdat=postdat[1:3,],
    SEM.pre = teststats$SEM.alpha)

##   pre.score post.score diff    SE      z     p
## 1        26         22   -4 7.026 -0.569 0.569
## 2         5          6    1 7.026  0.142 0.887
## 3        25         13  -12 7.026 -1.708 0.088
```
